# Medical Society of the State of New York

**Published:** 1908-03

**Authors:** Joseph D. Bryant, John G. Curtis

**Affiliations:** New York; New York


### CORRESPONDENCE

### Medical Society of the State of New York

**Committee on Experimental Medicine**

New York, January 21, 1908.

Editor Buffalo Medical Journal:

Sir—On April 8, 1907, the president of this society appointed twenty-four members thereof to be a "Committee on Experimental Medicine," in view of proposed legislation calculated to injure the progress of medicine by restricting experimentation.

As agitation in this direction has recently been renewed in a very plausible form, the undersigned have been instructed by the said committee to send you the following copy of a preamble and resolution adopted at a meeting thereof held in New York City, on January 15, 1908, and respectfully to request the publication thereof in the Buffalo Medical Journal:

Whereas, In the State of New York, a petition is being widely circulated among medical men for signature in favor of a proposed bill entitled "An act to prevent cruelty by regulating experiments on living animals"; and

Whereas, The said bill contains in its provisions conditions which would probably seriously impair the progress of scientific medicine,

Resolved, That the Committee on Experimental Medicine of the Medical Society of the State of New York earnestly requests the members of the medical profession to refrain from signing the aforesaid petition, and urges any who may have signed the same by inadvertence to withdraw their signatures.

The present laws of this State relating to this subject have long proved adequate and satisfactory.

Joseph D. Bryant, Chairman.
John G. Curtis, Secretary.

